# Screening for tuberculosis infection and effectiveness of preventive treatment among people with HIV in low-incidence settings

**DOI:** 10.1097/QAD.0000000000003747

**Published:** 2023-11-22

**Authors:** Dorine van Geuns, Rob J.W. Arts, Gerard de Vries, Ferdinand W.N.M. Wit, Svetlana Y. Degtyareva, James Brown, Manish Pareek, Marc Lipman, Reinout van Crevel

**Affiliations:** a Julius Centre for Health Sciences and Primary Care Medicine, University Medical Centre Utrecht, Utrecht; b Department of Internal Medicine and Radboud Center for Infectious Diseases, Radboud University Medical Center, Nijmegen; c National Institute for Public Health and the Environment (RIVM), Bilthoven; d Department of Internal Medicine, Division of Infectious Diseases, Amsterdam Institute for Infection and Immunity, Amsterdam University Medical Centers, University of Amsterdam; e Stichting HIV Monitoring, Amsterdam, the Netherlands; f Department of Infectious Diseases, Epidemiology and Phthisiology, RUDN University, Moscow, Russia; g Department of Respiratory Medicine, Royal Free London NHS Foundation Trust, London; h Department of Respiratory Sciences, University of Leicester; i Department of Infection and HIV medicine, Leicester Royal Infirmary, Leicester; j UCL Respiratory, University College London, London; k Centre for Tropical Medicine and Global Health, Nuffield Department of Medicine, University of Oxford, Oxford, UK.

**Keywords:** HIV, latent tuberculosis infection, meta-analysis, preventive therapy, screening, systematic review

## Abstract

**Objective:**

To determine the yield of screening for latent tuberculosis infection (LTBI) among people with HIV (PWH) in low tuberculosis (TB) incidence countries (<10 TB cases per 100 000 persons).

**Design:**

A systematic review and meta-analysis were performed to assess prevalence and predictive factors of LTBI, rate of TB progression, effect of TB preventive treatment (TPT), and numbers needed to screen (NNS).

**Methods:**

PubMed and Cochrane Library were searched for studies reporting primary data, excluding studies on active or paediatric TB. We extracted LTBI cases, odds ratios, and TB incidences; pooled estimates using a random-effects model; and used the Newcastle–Ottawa scale for bias.

**Results:**

In 51 studies with 65 930 PWH, 12% [95% confidence interval (CI) 10–14] had a positive LTBI test, which was strongly associated with origin from a TB-endemic country [odds ratio (OR) 4.7] and exposure to TB (OR 2.9). Without TPT (10 629 PWH), TB incidence was 28/1000 person-years (PY; 95% CI 12–45) for LTBI-test positive versus 4/1000 PY (95% CI 0–7) for LTBI-test-negative individuals. Among 625 PWH (1644 PY) receiving TPT, 15 developed TB (6/1000 PY). An estimated 20 LTBI-positive individuals would need TPT to prevent one case of TB, and numbers NNS to detect LTBI or prevent active TB varied according to a-priori risk of LTBI.

**Conclusion:**

The relatively high prevalence of LTBI among PWH and the strong correlation with origin from a TB-endemic country support risk-stratified LTBI screening strategies for PWH in low-incidence countries and treating those who test positive.

## Introduction

Globally, tuberculosis (TB) is a leading cause of death in people with HIV (PWH). TB preventive treatment (TPT) effectively prevents progression to TB disease among PWH [1], especially among those who screen positive for latent tuberculosis infection (LTBI) [2]. Therefore, the WHO recommends TPT for all PWH who screen positive, after exclusion of TB disease [3]. In low TB-incidence countries, testing for LTBI using tuberculin skin test (TST) and/or interferon gamma release assay (IGRA) is usually advised, often selectively to PWH deemed at higher risk such as immigrants [4]. However, a survey among low TB burden countries reported that only 75% had a national policy on LTBI, and that 66% provided LTBI testing and treatment for PWH [5]. The potential benefit of LTBI screening and TPT for PWH in low TB-incidence settings remains contentious [6], and adherence to these guidelines is low in many low-incidence countries such as the Netherlands [7], Belgium [8], the UK [9] and New Zealand [10]. One reason for this is that clinicians feel that PWH are at low risk for TB once effective antiretroviral therapy (ART) is started [7,11,12]. With the introduction of early and effective ART, the risk of TB has probably gone down over the years [13,14]. Still, also in TB low-endemic countries, many PWH present late with low CD4^+^ counts, and individual studies suggest that the risk of TB, even with early ART and proper immune reconstitution, remains higher for PWH than for people without HIV [15]. As such, the yield and outcome of LTBI screening and TPT for PWH in these settings is uncertain and it remains unclear what the benefit of screening and treatment of LTBI in HIV-infected patients in these settings is [6]. To address this knowledge gap, we conducted a systematic review and meta-analysis aiming to establish the LTBI prevalence and associated risk factors among PWH in low TB-incidence settings, and assess risk of progression to TB, and effectiveness of TPT.

## Methods

### Search strategy and selection criteria

Three separate research questions were addressed. Firstly, we wanted to establish the LTBI prevalence and associated risk factors among PWH in low TB-incidence settings (category: general, prevalence, predictive factors). Secondly, we wanted to assess the risk of progression to TB in LTBI in PWH in low TB-incidence settings (category: disease progression). Thirdly, we wanted to determine the effectiveness of TPT in LTBI in PWH on the progression to TB (category: prophylactic therapy). This third research question corresponds to the following PICO format. P: PWH with LTBI, I: TPT, C: no TPT, O: progression to TB.

We searched PubMed and Cochrane Library databases for relevant peer-reviewed studies (search performed 6 October 2020, rerun 9 December 2021). No filters were applied, and the full search strategies for the separate questions – indicated by corresponding category – are available in the supplementary material (Supplementary Table 1). References of relevant articles and guidelines were also checked. Studies were included if: full text was available in English or Spanish; the article reported primary data; the study was conducted in a low TB-incidence setting (<10 TB cases per 100 000 persons) [1]; if TST and/or IGRA data were reported; and if at least four PWH had been included in follow-up. Studies about active or paediatric TB were excluded. Search criteria and methods used are described in the research protocol (Supplementary Table 2).

### Data abstraction and quality assessment

Data were abstracted by one reviewer and included author, year, study design, setting, sample size, LTBI prevalence, method used to diagnose LTBI, demographic characteristics of the study population and HIV-related factors such as blood CD4^+^ cell count and ART status, person years (PY) of follow-up, and diagnosis of TB during follow-up. Quality assessment of data extraction was performed by two other authors. PRISMA-guidelines were followed for analysis and presentation of results. Quality assessment was performed using a tailored Newcastle–Ottawa scale to classify studies as low quality (<4 stars), moderate quality (4-5 stars), or high quality (>5 stars) (Supplementary Table 3)

### Data analysis and statistics

STATA version 16 was used for data processing, analyses, and producing figures. Heterogeneity was assessed for all meta-analyses using *I*^2^ statistic [16]. As there was substantial heterogeneity, all meta-analyses were carried out using a random-effects model. A funnel plot was used to assess small study effects. To assess LTBI prevalence, proportions of positive test results and total participants and their 95% confidence interval (CI) were pooled and presented in a forest plot with sub-analyses based on year of publication and originating continent. For each individual potential predictive factor, odds ratios and their 95% CI were calculated by test result or abstracted from the articles whenever available. Log-transformed odds ratios were pooled independently for every factor and back transformed. *P* less than 0.05 was used as a cut-off for significance.

Incidence of active TB during follow-up was assessed according to TST/IGRA test result and TPT. Incidence was expressed as the number of cases divided by person-years of follow-up whenever available, otherwise mean or median time of follow-up was used. To estimate progression rates, incidence rates of TB disease for each group were calculated per study. Subsequently, the incidence rate differences and ratios between these groups were calculated, which were then pooled with their 95% CI. Due to zero event studies, a continuity correction was applied. Studies with no events in either group were excluded from meta-analysis on incidence rate difference and ratio.

We calculated the number of individuals with a positive LTBI test that would need TPT to prevent one case of TB [number needed to treat (NNT)] as the inverse of the absolute risk reduction (ARR) assessed by our meta-analysis on the effectiveness of treatment [17]. Using pooled prevalence data, we also calculated the number needed to screen (NNS) to detect one case of LTBI stratified for predictive factors identified as described earlier. Finally, by dividing the NNT by the pooled prevalence, we estimated the NNS and NNT of PWH to prevent one case of TB, assuming those not screened would remain unaware of their LTBI status and all those with a positive test result would receive TPT.

## Results

### Search results

**Fig. 1:**
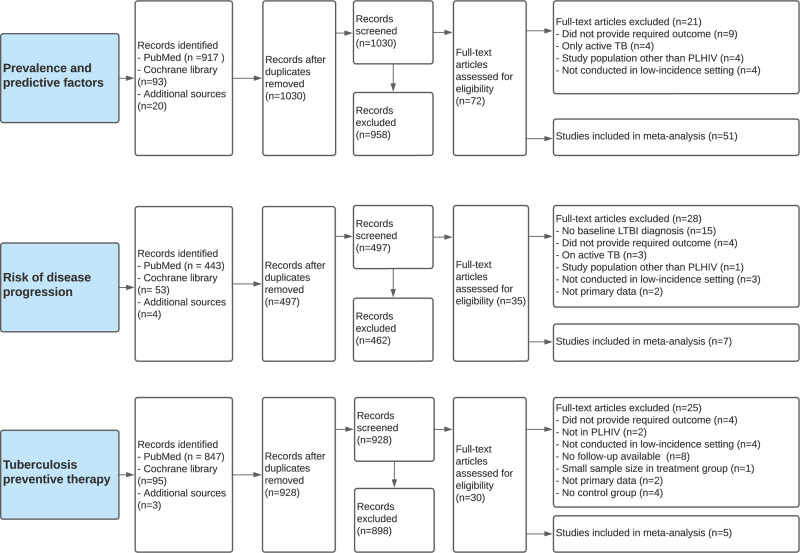
Article screening and selection.

After removal of duplicates, the search on the prevalence and predictive factors for LTBI among PWH in low TB-incidence settings yielded 1030 articles that were screened by title and abstract; 72 publications remained for full-text assessment and 51 were included in the analysis (Fig. 1). To assess TB disease progression, 497 articles were identified and screened by title and abstract, 35 were assessed by full-text and a total of seven were included in analysis. To examine the effectiveness of TPT, 928 articles were screened by title and abstract, 30 were eligible for full-text assessment and five were included in the analysis.

### Prevalence of latent tuberculosis infection and predictive factors for a positive test result

To assess LTBI prevalence, we included 39 cohort studies [10,18–55] and 12 cross-sectional studies [56–67], with a total of 65 930 PWH, as summarized in Table 1. Studies were published after 1991; they were conducted in Canada, the United States, Australia, New Zealand, and several Western and Southern European countries. These studies were aiming to either compare diagnostic test performance [43,48,63], provide data on TB and LTBI during routine medical check-ups [22,24,39,68], estimate the prevalence of LTBI [44], or evaluate LTBI screening and treatment [41]. Most studies excluded TB disease, either by clinical assessment [28], sputum smear and culture, or chest X-ray, conducted before or after LTBI testing [24,29,43,58,62]. Some only reported the number of positive LTBI screening test results [31,44]. Most studies (73%) provided TST results, fewer provided Quantiferon (QFT, 68%) and T.SPOT.TB (41%) data. Percentages of indeterminate test results varied from 0% [10] to 5.7% [18] for QFT and from 1.3% [43] to 14% [67] for T.SPOT.TB. Ten older studies, mostly among newly diagnosed patients, reported skin test anergy, ranging from 18% [66] to 63% [47]. Four other studies found that test positivity was inversely associated with CD4^+^ cell count [28,32,39,43].

**Table 1:** Individual studies included.

Author (year)	Setting	Study design	Study period	Mean national TB incidence during study period (per 100 000 people)	Study population	PWH (*n*)	Age at enrolment	CD4^+^ cell count at inclusion	On ART at inclusion (%)	HIV-1 RNA	LTBI test	LTBI prevalence (%)	Indeterminate (%)	Quality grading^b^
Aichelburg (2009)	Austria	Prospective cohort	2006–2008	10	PWH attending HIV unit	830	Median 39 (IQR 32–47)	Median 393 (IQR 264–566)	59.6%	Median 3.9 log (IQR 1.7–4.7)	QFT	4.5%	5.7%	Moderate
Anastos (1999)	United States	Prospective cohort	1994–1995	9	Women infected or at high risk of HIV	1343	36	<200 in 28%	34.5%	NA	TST	4.7%	NA	Moderate
Antonucci (2001)	Italy	Prospective cohort	1995–1998	9	PWH attending infectious disease unit	1215	Median 33 (IQR 18–75)	<200 in 30.9%	21.0%	NA	TST	6.7%	NA	High
Aston (2019)	United Kingdom	Retrospective cohort	2016–2017	10	PWH	25	Median 35.8 (IQR 28.3–45.0)	<200 in 25.2%	NA	NA	IGRA	8.0%	NA	Low
van Bentum (2018)	the Netherlands	Cross–sectional	2016–2017	6	PWH in outpatient clinic	599	NA	Mean 648	NA	NA	QFT	4.8%	0%	Moderate
Bourgarit (2015)	France	Prospective cohort	2009–2011	10	PWH	415	Mean 38.9 (±10.0)	Mean 483 (±242)	0%	Mean 4.2 log (±1.1)	QFT, T-SPOT.TB and TST	14.7%	2.4% (QFT) 5.5% (T-spot)	High
Brassard (2009)	Canada	Retrospective cohort	1988–2006	5	PWH attending HIV clinic	476	Mean 38.3 (R 14–72)	Mean 272 (R 0–1762)	26.3%	Mean 3.7 log (R 2.7–6.4)	TST	14.1%	NA	High
Brock (2006)	Denmark	Prospective cohort	2004–2005	8	PWH attending outpatient clinic	590	Median 43 (IQR 37–50)	Mean 523 (± 278)	75.9%	74% <500 copies/ml	QFT	4.6%	3.4%	High
Bua (2011)	Italy	Cross-sectional	NA	NA	Hospitalized PWH	73	Median 41 (IQR 21–63)	Mean 270 (R 4–897)	23%	Mean 5.8 log (r <40 to 15 000 000)	QFT	11.0%	16%	Moderate
Capocci (2020)	United Kingdom	Prospective cohort	2013–2020	10	PWH attending HIV clinic	217	Median 46 (IQR 41–52)	NA	NA	84% <50 copies/ml	T.SPOT.TB and TST	6.4%	NA	Moderate
CDC (1993)	United States	Prospective cohort	1990–1991	10	Persons attending drug-treatment centres and correctional facilities	1516	NA	NA	NA	NA	TST	18.9%	NA	Low
CDC (2000)	United States	Retrospective cohort	1998–1999	7	Close contacts of TB patients	95	NA	NA	NA	NA	TST	12.6%	NA	Low
Cheallaigh (2013)	Ireland	Cross-sectional	2008–2010	11	PWH attending HIV outpatient clinic	256	Median 36 (R 17–66)	Median 338 (R 1–1328)	69.1%	50% <50 copies/ml	T-SPOT.TB, QFT and TST	18%	2% (QFT) 7% (T-spot)	Moderate
Cordioli (2018)	Italy	Prospective cohort	2001–2017	8	PWH attending HIV outpatient clinic	717	NA	Mean > 500 cell/μl	NA	NA	QFT	5.3%	1.7%	Low
Daley (1998)	United States	Prospective cohort	1990–1994	10	Injection drug users	338	NA	NA	NA	NA	TST	28.0%	NA	Low
Diez (2007)	Spain	Prospective cohort	2000–2003	25	PWH attending HIV clinic	954	75% aged <40 years	NA	NA	8.6% <50 copies/ml	TST	21.3%	NA	High
Doyle (2014)	Australia	Retrospective cohort	2003–2011	6	PWH attending HIV clinic	917	Median 40.9 (IQR 33–48)	Median 491 (IQR 348–677)	NA	Median <50 copies/ml (IQR <50–1360)	QFT	3.2%	0.4%	High
Eriksen (1998)	United States	Cross-sectional	1996–1997	8	Women infected with HIV	159	Pregnant women (mean 22.9, SD 5.3), nonpregnant (mean 28.2, SD 7.8)	Pregnant women (mean 466, SD 258), nonpregnant (mean 456, SD 279)	NA	NA	TST	11.3%	NA	Low
Gampper (1998)	United States	Cross-sectional	1992–1994	10	PWH attending HIV outpatient clinic	341	68% aged between 25 and 40	NA	NA	NA	TST	10%	NA	Moderate
Girardi (2000)	Italy	Prospective cohort	1995–1998	NA	PWH attending infectious diseases hospital units	1360	Mean 34.3 (R 18–74)	<200 × 10^6^/l in 38.8%	34.7%	NA	TST	7.0%	NA	Moderate
Goletti (2020)	Italy	Retrospective cohort	2016–2017	7	Newly diagnosed PWH, cART na	495	Median 38 (IQR 30–48)	Median 319 (IQR 133–540)	0.0%	Median 4.9 log (IQR 4.3–5.4)	QFT or TST	6.5%	3.2%	Moderate
Golub (2008)	United States	Prospective cohort	1988– 2004	8	Injection drug users	800	NA	NA	NA	NA	TST	16%	NA	Moderate
Gow (2017)	New Zealand	Retrospective cohort	2002– 2014	10	PWH attending HIV outpatient clinic	752	Median 46	Median 343^a^	85.6%	66% <1.3 log copies/ml	QFT or TST	10%	0.0%	Moderate
Gourevitch (1998)	United States	Prospective cohort	1995–1996	9	Drug users	159	NA	NA	NA	NA	TST	16%	NA	Moderate
Ho (2022)	United States	Prospective cohort	2012–2020	3	People at high risk for LTBI	1873	NA	NA	NA	NA	QFT, T.SPOT.TB and TST	8.3%	NA	Moderate
Huebner (1994)	United States	Cross-sectional	1993	10	PWH attending HIV or tuberculosis clinic	479	Median 34	Median 349 (SD 291)	NA	NA	TST	12%	NA	High
Kall (2012)	United Kingdom	Cross-sectional	2006–2009	15	PWH attending HIV outpatient clinic	520	44% aged 35–44	Median 458 (IQR 312–631)	66.9%	NA	T.SPOT.TB	9.2%	3.5%	Moderate
Kusejko (2020)	Switzerland	Prospective cohort	1988 – 2020	5	PWH attending outpatient clinic	13 675	NA	NA	NA	NA	IGRA and TST	6.1%	NA	Moderate
Lee (2006)	United States	Prospective cohort	1995–1997	8	HIV/AIDS reported cases	869	NA	NA	82.7%	NA	TST	6.7%	NA	High
Lobato (2003)	United States	Prospective cohort	1993–1996	9	Inmates in correctional facilities	4435	Median 30 in complete study	NA	NA	NA	TST	42%	NA	Low
Luetkemeyer (2007)	United States	Cross-sectional	NA	NA	ART-treated patients and indigent adults	294	Median 46 (IQR 42–51)	Median 363 (IQR 214–581)	68.7%	Median 2.4 log (IQR 2.0–4.4)	QFT and TST	12.2%	5.1%	Moderate
Lyne (2013)	Australia	Cross-sectional	2009–2010	7	PWH attending sexual health services	240	Median 47 (R 13–79)	NA	NA	NA	IGRA	7.9%	1.3%	Moderate
Marks (2008)	United States	Cross-sectional	2004	5	Ballroom community	83	67% aged between 15 and 24 in complete study	NA	NA	NA	TST	3.6%	NA	Low
Markowitz (1993)	United States	Prospective cohort	1988–1990	9	HIV-seropositive and HIV-seronegative persons	1171	Mean 37 (R 18–67)	Mean 438 (SD 270)	NA	NA	TST	5.5%	NA	High
Martínez-Pino (2013)	Spain	Prospective Cohort	2004–2009	17	PWH attending hospital	2540	Median 38 (IQR 32–43)	Median 415 (IQR 255–611)	8.1%	Median 80 (IQR 49–15,988)	TST	16.9%	NA	High
Mofenson (1995)	United States	Prospective cohort	1989 – 1993	10	Women living with HIV	183	54% aged 20–29	NA	NA	NA	TST	14%	NA	Moderate
Narita (2002)	United States	Prospective cohort	1999–2001	6	PWH attending HIV clinic	2127	NA	NA	NA	NA	TST	8.4%	NA	Moderate
Pascopella (2014)	United States	Retrospective cohort	2008–2010	4	PWH attending HIV clinic	393	Median 46 (R 18–86)	Median 437 (R 2–1458)	96%	Median 50 (R 0–5 410 000)	QFT, TST	5%	2	High
Pullar (2014)	Norway	Prospective cohort	2009–2012	8	PWH attending outpatient clinic	304	NA	NA	65.1%	NA	QFT, T.SPOT.TB and TST	21%	1.0% (QFT) 1.3% (T-spot)	Moderate
Reaves (2017)	United States	Retrospective cohort	2010–2012	4	PWH attending outpatient clinic	1907	27% aged 40–49	NA	86.1%	52% <200 copies/ml	TST or QFT	6.9%	1.2%	Low
Sackoff (1998)	United States	Retrospective cohort	1995–1998	8	PWH attending outpatient HIV clinic	757	Median 38	48% <200	NA	NA	TST	6%	NA	Moderate
Sandhu (2020)	United Kingdom	Prospective cohort	2018–2019	8	PWH attending outpatient HIV clinic	128	Mean 47	Median 352 (IQR 187–586)	NA	Mean 2.3 log copies/ml (SE 0.1)	QFT	2.3%	3.1%	Low
Scholten (2003)	United States	Prospective cohort	1993–1998	9	Injection drug users	373	Mean 40 (R 21–67) in whole study	32% <200	NA	NA	TST	37.8%	NA	Moderate
Schulte (2002)	United States	Cross-sectional	1995–1996	9	Pregnant, HIV-infected women	207	Median 26 (R 15–45)	NA	NA	NA	TST	26%	NA	Moderate
Sester (2016)	Europe	Prospective cohort	2008–2013	NA	Immunocompromised patients attending healthcare facilities	768	Median 41 (IQR 34–48)	Median 302 (IQR 196–370)	NA	NA	QFT, T.SPOT.TB and TST	15.9%	3.1% (QFT) 11.2% (T-spot)	High
Shin (2016)	United States	Retrospective cohort	2010–2013	4	PWH attending HIV clinic	15346	35% aged 40–49	NA	NA	NA	QFT and TST	5.3%	2.8%	High
Snyder (1999)	United States	Prospective cohort	1990–1998	9	People attending to methadone maintenance clinic	472	Median 40 (R 18–77) in complete study	NA	NA	NA	TST	23.5%	NA	Moderate
Stephan (2008)	Germany	Prospective cohort	2006–2007	7	PWH attending outpatient HIV department	275	Median 44 (R 22–75)	Median 408 (R 7–1510)	83.2%	Median 1.6 log (R 1.6–6.9)	QFT, T.SPOT.TB and TST	36.6%	0.4% (QFT) 2.9% (T-spot)	Moderate
Stout (2018)	United States	Prospective cohort	2012–2014	4	Children and adults at high risk for LTBI	1366	Foreign-born: median 46 (IQR 38–56), US-born: median 50 (IQR 43–55)	NA	NA	NA	QFT, T.SPOT.TB	12.7%	NA	Moderate
Talati (2009)	United States	Cross-sectional	2005–2006	6	PWH attending outpatient HIV clinic	336	Mean 42 (R 22–79)	Median 334 (R 0–1380)	68.8%	Median 400 (R <50 - > 5.9 log)	QFT, T.SPOT.TB and TST	8%	1.8% (QFT) 14% (T-spot)	High
Trieu (2015)	United States	Prospective cohort	2005–2014	5	Contacts of persons infected with multidrug-resistant tuberculosis	167	NA	NA	NA	NA	TST	3.0%	NA	Low

ART, antiretroviral therapy; cART, combination antiretroviral therapy; IGRA, interferon gamma release assay; LTBI, latent tuberculosis infection; NA, not available; PWH, people with HIV; QFT, QuantiFERON-TB; TST, tuberculin skin test.

a Range or IQR not reported.

b Done according to the STARD-checklist, with the following classification: reporting less than 10 items as low quality, 10–16 items as moderate quality and more than 16 as high quality. NA, not applicable. The decision on inclusion of low-incidence countries (<10 TB cases per 100 000 population) was based on the year of publication of the study. In some countries, for example, the United Kingdom, the TB incidence was greater than 10/100 000 at the time of study but had progressed to the low-incidence state at the time of publication. We kept these studies to allow sufficient number of publications for analysis.

The median age of patients ranged from 36 [58,68] to 46 [10,63] years. Most studies had an overrepresentation of PWH originating from TB-endemic countries, varying from 22% [53] to 72% [43]. Also, PWH with low CD4^+^ cell counts were more likely to be screened for LTBI [31,68]. LTBI screening was performed less often among those on successful ART [68]. Median baseline CD4^+^ cell counts, reported in 16 of 22 studies, ranged from 272 [23] to 648 [56] at enrolment; six studies reported proportions of those with less than 200 CD4^+^ cells, ranging from 25% [21] to 48% [45]. Median plasma HIV-RNA (reported in 14 studies) ranged from less than 50 copies/ml [29,53] to 4.9 log_10_ copies/ml [31]. Use of ART, reported in 14 studies, ranged from 0% [22] to 86% [44], but only 10 studies reported the proportion of patients with virological suppression, ranging from 0% [48] to 72% [44]. Eight out of 17 studies with data on date of HIV diagnosis had only included newly diagnosed patients; in the other studies, time between HIV diagnosis and LTBI screening ranged from less than 3 years [45] to a median of 8.6 years [42]. Further systematic analysis of LTBI screening in relation to time of HIV diagnosis, CD4^+^ cell count, ART use, and plasma HIV-RNA was not possible as individual data were not reported.

**Fig. 2:**
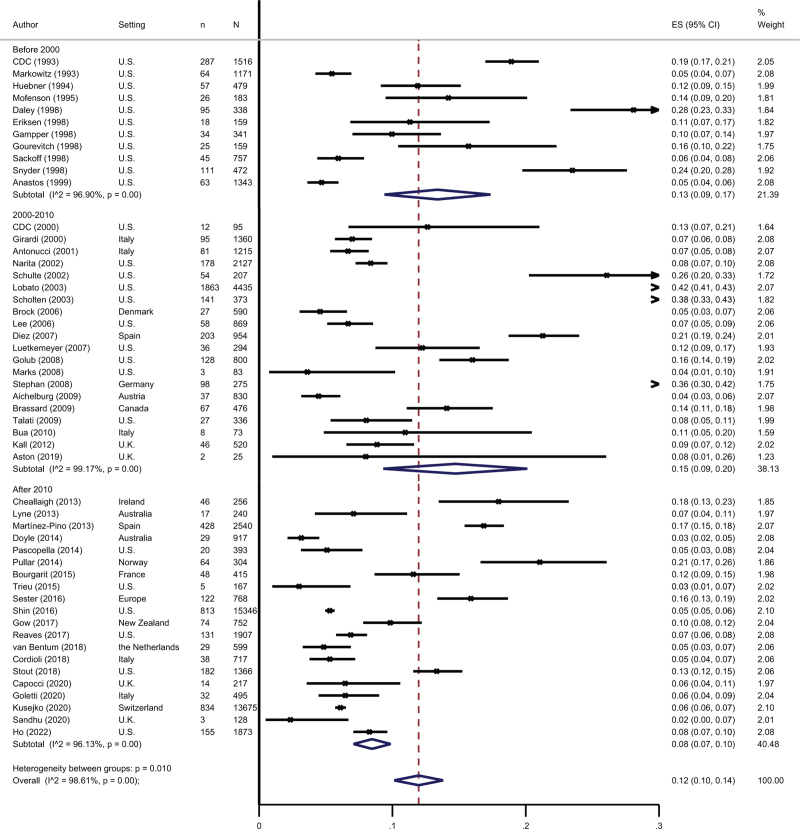
Forest plot of latent tuberculosis infection prevalence among people with HIV in low tuberculosis-incidence settings stratified by year of study publication.

The random effects meta-analysis resulted in a pooled LTBI prevalence of 12% (95% CI 10–14%), with a trend towards lower prevalence in more recent studies (Fig. 2). Prevalence also varied by region (Supplementary Figure 1). The funnel plot showed some evidence for small-study effects (Supplementary Figure 2).

Positive LTBI test results were significantly associated with ethnicity or geographical region of origin of PWH, as well as TB exposure. Many studies reported higher LTBI prevalence in people originating from TB-endemic countries (pooled odds ratio 4.7; 95% CI 3.7–5.8) [18,22–24,29,53,62,63,68], or sub-Saharan African origin (OR 3.3; 95% 1.0–10.2) [18,39,62] (Table 2). Exposure to a patient with TB or long-term stay in a high-incidence setting was also associated with test positivity (OR 2.9; 95% CI 2.0–4.3) [22,24,28,43,58,63,64,66]. In our meta-analysis, gender, injection drug use, CD4^+^ cell count, ART and viral load were not significantly associated with a positive test result, with the following corresponding OR for male individuals (OR 0.65, 95% CI 0.40–1.05, *P* value 0.08) and ART use (OR 0.55, 95% CI 0.29–1.04, *P* value 0.06).

**Table 2:** factors associated with a positive latent tuberculosis infection test result.

Predictive factor	Number of studies	*n*/*N*	Pooled odds ratio (95% CI)^b^	Test for overall effect	Test for heterogeneity
				*P* value	*I* ^2^
Gender (male)	16	40 342/49 928	0.65 (0.40–1.05)	0.08	95.0%
Origin from TB-endemic country	11	2582/12 874	4.67 (3.74–5.84)	<0.001	0.0%
Sub-Saharan African^a^	4	852/6280	3.26 (1.04–10.17)	0.04	84.3%
African-American^a^	4	3958/8242	1.77 (0.57–5.51)	0.3	88.9%
Foreign born (yes)	8	7521/21 860	3.28 (1.95–5.53)	<0.001	89.9%
Injecting drug use	13	4756/22 394	1.08 (0.74–1.6)	0.70	70.1%
Heterosexual HIV transmission vs. MSM	7	8686/13 093	1.85 (1.17–2.93)	0.01	82.1%
Exposure to TB^c^	8	162/3,317	2.88 (1.96–4.25)	<0.001	27.3%
CD4 (<200)	9	2574/10 338	0.82 (0.40–1.68)	0.59	86%
ART (yes)	9	6196/18 140	0.55 (0.29–1.04)	0.06	86.7%
Viral load (<50 copies/ml)	4	710/2,524	0.93 (0.54–1.62)	0.81	70.2%

ART, antiretroviral therapy; CI, confidence interval *N*, number of available data; *n*, number of people with factor of interest.

a African-American in US studies and participants with origin in sub-Saharan Africa in European studies.

b Derived from random-effects meta-analysis.

c TB contact or previous TB exposure.

### Risk of disease progression in the absence of tuberculosis preventive treatment

Incidence rates were used from seven cohorts (10 629 PWH at risk during 92 915 person-years) to calculate the risk of progression (Supplementary Table 4). Combining all studies, a total of 133 PWH (1.25%) were diagnosed with TB during follow-up. Three studies reported disease localization; pulmonary TB was diagnosed in 57–91% of patients [18,39,68]. Incidence rates in those with a positive TST or IGRA at time of inclusion ranged from 12.7 [48] to 48.4 cases [18] per 1000 person-years, and from 0 [18,43] to 10.4 per 1000 person-years [39] in those with a negative TST or IGRA. The pooled incidence rate derived from random-effects meta-analysis was 28 per 1000 person-years (95% CI 12–45) for TST/IGRA-positive and 4 cases per 1000 person-years (95% CI 0–7) for TST/IGRA-negative individuals (Table 3). This represented a pooled incidence rate ratio of 8.8 (95% CI 3.7–20.7).

**Table 3:** Tuberculosis incidence according to baseline TST or IGRA test result.

Outcome	No. studies	Total no. of patients at risk	Total no. of cases	Total person years at risk	Pooled incidence rate per 1000 PY (95% CI)^a^	Test for overall effect	Test for heterogeneity
						*P* value	*I* ^2^
Overall incidence	7	10 629	133	31 141	4 (0–7)	0.07	50.3%
PWH with a positive baseline TST or IGRA	7	953	60	2218	28 (12–45)	0.001	100%
PWH with a negative baseline TST or IGRA	7	9676	73	28 923	4 (0–7)	0.05	100%
Incidence rate difference	7	10 629	133	31 141	29 (16–41)	<0.001	98.9%
Incidence rate ratio	6	10 025	133	30 633	8.77 (3.71–20.73)^b^	0.003	72.7%

CI, confidence interval; IGRA, interferon gamma release assay; PWH, people with HIV; PY, person-years; TST, tuberculin skin test.

a Derived from random-effects meta-analysis.

b Incidence rate ratio is not expressed per 1000 PY, but as a ratio.

Two studies found a significantly higher risk of TB disease for PWH with a low CD4^+^ cell count [39,69]. One study reported a relatively low median CD4^+^ cell count (208 cells/μl) at the time of TB diagnosis [68]. The proportion of patients on ART at enrolment, reported in four studies, varied, and ranged from 40.8% [68] to 100% [69]. Five studies found that disease progression was significantly more common in PWH not on ART and/or with detectable plasma HIV-RNA [18,29,48,68,70]. However, specific analysis of TB risk according to CD4^+^ cell count or use of ART could not be done in the absence of individual data.

### Effectiveness of tuberculosis preventive treatment

Our literature search identified four cohort studies [23,39,43,68] and one randomized controlled trial [71], which reported on the effectiveness of TPT in PWH with a positive LTBI test (Supplementary Table 5). In these studies, published between 2007 and 2014, a total of 625 test-positive PWH (range: 67–411 for individual studies) who received TPT were followed for 1020 person-years (range: 78–648 person-years), while 469 test-positive individuals (range: 15–246) who did not receive TPT were followed for 1423 person-years (range: 50–1005 person-years). One study lacked a control group [72] and three studies reported no cases in the treatment group [23,43,68] of which one [47] had no TB cases in either arm during 128 person-years of follow-up, so a continuity correction was applied for these groups. The study without TB cases in either group [43] and the one without a control group [72] were excluded from meta-analysis on incidence rate difference and ratio.

Incidence rates between individual studies ranged from 0 to 17.5 cases per 1000 person-years for those who received TPT, and from 0 to 183.5 for those who did not. The pooled incidence rate derived from random-effects meta-analysis in the TPT group was 6 cases per 1000 person-years (95% CI 5–7). The pooled incidence rate in the non-TPT group was 65 cases per 1000 person-years (95% CI 26–103). The pooled absolute incidence rate difference was 44 (95% CI 20–94) The pooled incidence rate ratio was 0.02 (95% CI 0.00–0.89) (Supplementary Table 6). Few data were available for further analysis of risk factors, but one study reported a higher rate of TB despite TPT for individuals with a nadir CD4^+^ count of less than 200 cells/μl or of African ethnicity, the latter possibly due to newly diagnosed infections [39]. No study reported TPT stratified by ART status.

### Numbers needed to screen and treat

Based on the ARR of 0.05 (−0.04 to 0.15) derived from meta-analysis of the effectiveness of TPT, the estimated number of LTBI-positive individuals at inclusion needing TPT to prevent one case of TB (NNT) was 20 (95% CI 16–27) (Supplementary Table 7). Finally, using the estimate of 12% LTBI prevalence, the overall NNS of PWH to detect one case of LTBI was 8.3 (95% CI 8.2–8.5); ranging from 4 to 14 stratified by risk group). Presuming 100% uptake of TPT, the NNS of PWH to prevent one case of TB was 167 (95% CI 155–180), ranging from 111 for foreign-born individuals to 285 for native-born persons in low-incidence countries (Supplementary Table 7).

## Discussion

Our systematic review and meta-analysis revealed that one in eight PWH living in low TB-incidence countries who were screened for LTBI tested positive. LTBI prevalence was strongly associated with origin from a TB-endemic country, sub-Saharan African ethnicity, and close TB contact. Second, our study showed that the risk for TB progression was seven times higher among PWH with a positive LTBI test, while risk of TB appeared to be lower among individuals with higher CD4^+^ cell counts and those using ART. Third, TPT effectively prevented progression to TB in PWH with LTBI. These findings suggest that a risk-stratified approach of LTBI screening among PWH in low TB-incidence countries, with TPT for all those testing positive, may be appropriate in these settings.

LTBI prevalence varied between studies, most likely because of differences in epidemiological background of PWH included, and differences in LTBI screening strategies and tests. It is well known that TST and IGRA lack concordance, and both poorly reflect the risk of progression to TB [73]. Furthermore, test sensitivity is reduced in advanced HIV disease and low CD4^+^ cell counts, with more frequent indeterminate IGRA tests reported [74]. Positive LTBI tests were more common among PWH from TB-endemic countries or sub-Saharan Africa, as noted previously [75], though few studies stratified results among foreign-born according to TB burden in the country of origin [18].

We found that the risk of TB in PWH was seven times higher among individuals with a positive LTBI test than in those with a negative test. As the overall incidence appeared approximately 100-fold higher than the TB incidence in low-incidence countries (<10 cases per 100 000 persons), screening appears favourable in this population. The TB risk for PWH on ART may be lower nowadays as current international guidelines recommend PWH to start ART shortly after diagnosis of HIV, irrespective of their CD4^+^ cell count [15]. In an analysis that also included data from medium TB-incidence countries (defined as <100/100 000 person-years) the incidence appeared to have decreased more recently [76]. Although early and effective ART preserves and restores anti-TB-specific immune responses in PWH, the risk of progression from LTBI to TB remains higher than in people living without HIV [48].

We found TPT to be very effective in PWH with LTBI as it led to an approximately 90% reduction in TB incidence. Numbers of PWH NNS to detect one case of LTBI and numbers NNT to prevent one case of TB varied between studies and the NNS and NNT derived from our meta-analyses should be interpreted with caution because of the pooling of data [77]. As our analysis was based on a meta-analysis of only three studies regarding TPT, all reporting very few TB cases and relatively short follow-up, confidence intervals in our analysis were large, which demonstrates another limitation. Also, for our estimations, we assumed that all test-positive individuals would receive TPT, although a recent cascade-of-care analysis reported that among LTBI test-positive PWH in low-endemic settings, a pooled 86.3% initiated TPT [12]. Lastly, adherence and TPT completion was not evaluated in all studies, which could also result in an underestimation of the effectiveness of TPT.

Our study was limited by the heterogeneity between studies, inclusion of small studies, selective inclusion of patients, and a lack of individual patient data on ART, CD4^+^ cell count, and other determinants in included studies. Included studies suffered from selection bias, with an overrepresentation of individuals at increased risk for LTBI (because of migrant status or social determinants, and TST more often performed in these groups) and likely overestimation of LTBI prevalence among PWH in low TB-incidence settings [10,18,23,68]. Misclassification because of self-report may have led to inappropriate inclusion of individuals previously treated for TB or LTBI. Higher anergy rates in older studies may reflect suboptimal uptake of ART or poor control of HIV, which would underestimate the LTBI prevalence. The meta-analysis was influenced by sparse data bias because of low TB incidence in these settings, which makes it harder to assess risk factors of TB progression. Moreover, the time between HIV diagnosis and assessment of LTBI, and the time to TB diagnosis was not always available, which also complicates interpretation. Due to applying a continuity correction for studies that had zero events in one group in combination with low incidence rates in other groups, we might have overestimated the rate of disease progression and underestimated the effectiveness of preventive treatment.

Our results show that TB remains more common among PWH than the general population in low TB-incidence settings and that TPT can effectively prevent TB. However, it should be noted that this result may be overestimated because of the inclusion of those more at risk for TB and by inclusion of countries that used to have a higher TB incidence during the study period compared with now. Our findings suggest that targeted LTBI screening among PWH in low TB-incidence countries could be more efficient and cost-effective than current strategies, as suggested by a recent interventional study [78]. Certain groups among PWH could be prioritized for screening, such as those from TB-endemic countries or with a history of exposure. This should be accompanied by interventions to increase uptake of TPT for people with positive LTBI test results, especially among those deemed at the highest risk for developing TB [79].

Current guidelines vary between countries, and adherence to these guidelines is often poor [4,7–11]. Some low TB-incidence countries have adopted the WHO recommendation to screen all PWH for LTBI, whereas others use more selective screening approaches [6,80,81]. However, nowadays people with newly diagnosed HIV usually immediately start potent ART, resulting in faster restoration of CD4^+^ counts, leading to lower TB risk (irrespective of LTBI status) but with still significantly increased TB incidences [15,82]. Future studies should establish the effectiveness and cost utility of targeted LTBI screening among PWH in low TB-incidence countries where there is widespread ART use; and promote strategies to improve uptake and completion of TPT among those with positive LTBI tests.

## Acknowledgements

R.v.C. and G.d.V. have conceived the study, D.d.G. has performed the literature search and primary analysis, which was assessed for quality by R.v.C. and R.A. Statistical analysis was performed by G.d.V. with consultation of G.d.V. and F.W.N.M.W. All authors contributed to data analysis and interpretation. D.d.G. and R.v.C. have written the first draft, to which all commented; R.A. has reworked the final draft.

### Conflicts of interest

There are no conflicts of interest.

## Supplementary Material
