# Supplementary material for: Screening for tuberculosis infection and effectiveness of preventive treatment among people with HIV in low-incidence settings
Source: AIDS. 2023 Nov 22;38(2):193–205. doi: 10.1097/QAD.0000000000003747 (PMC10734787; doi:10.1097/QAD.0000000000003747)
Supplement: Supplementary file 1 [file aids-38-193-s001.docx]

Supplementary table 1: search strategy

| Date searched | Category | Number of results | Database | Search terms | Filters applied |
| --- | --- | --- | --- | --- | --- |
| 6^th^ October 2020, rerun 9^th^ December 2021 | General, prevalence, predictive factors | 822 | PubMed | ((latent TB [tiab]) OR (latent tuberc* [tiab]) OR (inactive tuberc* [tiab]) OR (LTBI [tiab]))  AND ((HIV [tiab]) OR (human immunodeficiency [tiab]))  AND ((low-inciden*) OR (low-burden) OR (high-income) OR (resource-rich) OR (screening) OR (programme) OR (control) OR (prevalence) OR (epidemiology) OR (diagnos*) OR (guideline*)) | none |
| 6^th^ October 2020, rerun 9^th^ December 2021 | Disease progression | 389 | PubMed | ((latent TB [tiab]) OR (latent tuberc* [tiab]) OR (inactive tuberc* [tiab]) OR (LTBI [tiab]))  AND ((HIV [tiab]) OR (human immunodeficiency [tiab]))  AND ((rever*) OR (conver*) OR (recurren*) OR (re-activat*) OR (inciden*) OR (re-infection) OR (progress*) OR (trends)) | none |
| 6^th^ October 2020, rerun 9^th^ December 2021 | Prophylactic therapy | 744 | PubMed | ((latent TB [tiab]) OR (latent tuberc* [tiab]) OR (inactive tuberc* [tiab]) OR (LTBI [tiab])) AND ((HIV [tiab]) OR (human immunodeficiency [tiab])) AND ((therapy) OR (treatment) OR (prevent*) OR (prophyla*) OR (isoniazid) OR (rifapentine) OR (number needed to treat) OR (NNT) OR (medication)) | none |
| 6^th^ October 2020, rerun 9^th^ December 2021 | General, prevalence, predictive factors | 128 | Cochrane | (((latent tuberculosis) OR (latent TB) OR (inactive tuberculosis) OR (LTBI))):ti,ab,kw AND ((HIV) OR (human immunodeficiency)):ti,ab,kw AND ((low-incidence) OR (low-burden) OR (high-income) OR (resource-rich) OR (screening) OR (programme) OR (control) OR (prevalence) OR (epidemiology) OR (diagnosis) OR (guideline)):ti,ab,kw | none |
| 6^th^ October 2020, rerun 9^th^ December 2021 | Disease progression | 67 | Cochrane | (((latent tuberculosis) OR (latent TB) OR (inactive tuberculosis) OR (LTBI))):ti,ab,kw AND ((HIV) OR (human immunodeficiency)):ti,ab,kw AND ((reversion) OR (conversion) OR (recurrence) OR (re-activation) OR (incident) OR (incidence) OR (re-infection) OR (progression) OR (trends)):ti,ab,kw | none |
| 6^th^ October 2020, rerun 9^th^ December 2021 | Prophylactic therapy | 130 | Cochrane | (((latent tuberculosis) OR (latent TB) OR (inactive tuberculosis) OR (LTBI))):ti,ab,kw AND ((HIV) OR (human immunodeficiency)):ti,ab,kw AND ((therapy) OR (treatment) OR (prevention) OR (prophylactic) OR (prophylaxis) OR (isoniazid) OR (preventive) OR (rifapentine) OR (number needed to treat) OR (NNT) OR (medication)):ti,ab,kw | none |

Supplementary table 2: research protocol

| Title | Screening for latent tuberculosis among HIV-patients in low-incidence settings |
| --- | --- |
| Background | TB is still the leading cause of death among people living with HIV. HIV may increase the risk of infection with TB following exposure and accelerates the progression of latent TB infection to disease with incidence of disease 20 times higher than HIV uninfected individuals. According to the WHO, the percentage of notified TB patients who had a documented HIV test result in 2018 was 64%.  The WHO made a recommendation to screen for latent tuberculosis infection among HIV-patients. However, this advice is not put into practice in many low-incidence settings. There is an effective treatment for HIV and exposure to tuberculosis is relatively low, which reduces the risk of progression from the latent to the active form of TB. There are various risk groups that could be screened for LTBI. For example, in most low-incidence countries, migrants from areas with a high TB burden make up the majority of the cases.  There are two types of tests available for screening for LTBI: the tuberculin skin test and the IGRA’s. The sensitivity and specificity are controversial, which also affects the cost-effectiveness of screening procedures. |
| Objectives | 1. Summarize studies describing screening for LTBI among HIV-patients in low-incidence settings 2. Describe the advantages and disadvantages of screening within this population and these settings considering:   The prevalence of HIV and tuberculosis  The instruments used for screening and their specificity, sensitivity and predictive value  The health benefits of prophylactic treatment of LTBI  The cost-effectiveness of screening procedures and available tests  The exposure to TB and risk groups  Determinants of progression from active TB to latent TB   1. Identify the strengths and weaknesses in these studies 2. Identify priority areas for further research 3. To identify and make recommendations for screening procedures for LTBI among HIV-patients in low-incidence countries |
| Methods | *Study Design:* Systematic review and meta-analysis  *Search criteria*  A search of the PubMed and Cochrane library databases will be done using the following key words:    Latent tuberc*, latent TB, LTBI, HIV, low-inciden*, low-burden, screening/diagnosis, guidelines, tuberculin test, interferon-gamma/γ, release test/assay, number needed to treat/screen, preventive treatment/therapy, prevention, immunosuppress*, tuberculosis control, costs/cost-effectiveness, re-activation/recurrence/progression/re-infection  In addition, experts in the field were contacted for additional publications. Also, references of pertinent articles were considered.  *Inclusion and Exclusion criteria:*  In order to be included in the review, studies have to:   - Include description of study design, methodology and statistical analysis - Consider latent tuberculosis - Have been conducted in low TB incidence settings - Have been published after January 1^st^ 1991 - Provide a full-text available in English or Spanish   Review articles will be excluded from analysis.  *Data extraction*  Titles and abstracts and from publications retrieved from initial search will be stored into a database. The abstracts will be screened for eligibility. Articles of abstracts are considered eligible after initial screen will be retrieved and data extracted using a data abstraction form. The following data elements will be abstracted onto forms and entered into an Excel spreadsheet.   - Year of publication - Author(s) - Title - Abstract - Keywords - Inclusion or exclusion and reasons - location (setting, country, region) where study was conducted - objectives of the study - study design - main findings - limitations of study - Data on LTBI prevalence, method used to diagnose, predictive factors, tuberculosis incidence, time to follow-up and treatment outcomes |
| Findings/ Results | Findings of the review will be analyzed and presented as follows:   1. Description of studies:   Number of studies identified, number of studies that were eligible/ not eligible by location and date of publication, location   1. Analyses on prevalence, predictive factors, disease progression, prophylaxis 2. Numbers needed to screen and needed to treat as a result of the meta-analyses |
| *Discussion/conclusion* | Strengths of study  Limitations of study  Risk of bias  Implications and recommendations for screening policies  Suggestions for further research |

Supplementary table 3: tailored Newcastle-Ottowa scale

| Criteria | 1 star | No star |
| --- | --- | --- |
| Is active TB excluded? | Yes | No, no description |
| Representativeness of the study population | Truly representative, somewhat representative | Selected group, no description |
| Was screening complete? | Complete, unlikely to cause bias | Possible differential screening |
| Ascertainment of exposure | Secure record, structured interview | Self-report, no description |
| Assessment of outcome | Independent blind assessment, record linkage | Self-report, no description |
| Adjust for the most important risk factors | Age, sex | No, no description |
| Adjust for other risk factors | Origin, smoking, alcohol, etc. | No, no description |

Supplementary table 4: study characteristics, outcomes and quality grading for included studies on disease progression

| No. | Author (year) | Setting | Study design | Positive TST or IGRA at time of inclusion | | | Negative TST or IGRA at time of inclusion | | | Incidence rate difference (95% CI) | Incidence rate ratio (95% CI) | Quality grading |
| --- | --- | --- | --- | --- | --- | --- | --- | --- | --- | --- | --- | --- |
|  |  |  |  | **No. of people at risk** | **Total person**  **years of**  **follow-up** | **Incidence rate per 1000 PY** | **No. of people at risk** | **Total person years of follow-up** | **Incidence rate per 1000 PY** |  |  |  |
| 1 | Aichelburg (2009) | Austria | Prospective cohort | 36 | 62 | 48.4 | 736 | 1169 | 0 | 0.05 (-0.01-0.10) | ∞ (7.79 -∞) | Moderate |
| 2 | Doyle (2014) | Australia | Retrospective cohort | 4 | 57 | 17.5 | 884 | 1733 | 0.6 | 0.02 (-0.02-0.05) | 30.40 (0.39-2386.58) | High |
| 3 | Elzi (2007) | Switzerland | Prospective cohort | 246 | 1005 | 15.9 | 3744 | 18,516 | 0.5 | 0.02 (0.01-0.02) | 29.48 (12.58-72.67) | Moderate |
| 4 | Martín-Echevarria (2011) | Spain | Retrospective cohort | 339 | 444 | 42.8 | 1485 | 1554 | 10.3 | 0.03 (0.01-0.05) | 4.16 (2.02-8.64) | Moderate |
| 5 | Martínez-Pino (2013) | Spain | Prospective cohort | 183 | 300 | 60 | 2031 | 3929 | 10.4 | 0.05 (0.02-0.08) | 5.75 (3.11-10.23) | High |
| 6 | Pullar (2014) | Norway | Prospective cohort | 57 | 114 | 0 | 197 | 394 | 0 | 0 | 0 | Moderate |
| 7 | Sester (2016) | Europe | Prospective and cross-sectional | 88 | 236 | 12.7 | 599 | 1627.5 | 3.1 | 0.01 (0.00-0.02) | 4.14 (0.64-21.3) | High |

Supplementary table 5: study characteristics, outcomes and quality grading for included studies on tuberculosis preventive treatment

| No. | Author (year) | Setting | Study design | Treatment completion rate | Treatment group | | | Control group | | | Incidence rate difference (95% CI) | Incidence rate ratio (95% CI) | Quality grading | |  |
| --- | --- | --- | --- | --- | --- | --- | --- | --- | --- | --- | --- | --- | --- | --- | --- |
|  |  |  |  |  | **No. of people at risk** | **Total person**  **years of**  **follow-up** | **Incidence rate per 1000 PY** | **No. of people at risk** | **Total person years of follow-up** | **Incidence rate per 1000 PY** |  |  | |  | |
| 1 | Brassard (2009) | Canada | Retrospective cohort | 77.6% | 52 | - | 0 | 15 | - | 183.5 | - 0.10 (-0.20-0) | ∞ | | High | |
| 2 | Elzi (2007) | Switzerland | Prospective cohort | 74.6% | 144 | 624 | 0 | 246 | 1005 | 15.9 | -0.01 (-0.02-0) | ∞ | | Moderate | |
| 3 | Martínez-Pino (2013) | Spain | Prospective cohort | 63.2% | 228 | 457 | 17.5 | 183 | 300 | 60 | -0.04 (-0.07- -0.01) | 0.29 (0.11-0.71) | | High | |
| 4 | Pullar (2014) | Norway | Retrospective cohort | - | 39 | 78 | 0 | 25 | 50 | 0 | 0 | - | | Moderate | |
| 5 | Rivero (2007) | Spain | Randomized controlled trial | 63% | 324 | 648 | 11.2 | - | - | - | - | - | | Moderate | |

Supplementary table 6: Tuberculosis incidence among LTBI-test positive PLHIV, according to tuberculosis preventive treatment (TPT) status

| Outcome | Studies (n) | patients at risk (n) | person years (PY) at risk | TB cases (n) | Pooled incidence rate per 1000 PY (95% CI)^§^ | Test for overall effect | Test for heterogeneity |
| --- | --- | --- | --- | --- | --- | --- | --- |
|  |  |  |  |  |  | **P-value** | **I^2^** |
| Overall TB incidence rate per 1000 PY | 5 | 1,094 | 3,032 | 53 | 10 (1-19) | <0.001 | 94.3% |
| Incidence rate per 1000 PY for those receiving TPT | 5 | 625 | 1644 | 15 | 6 (5-7) | <0.001 | 100% |
| Incidence rate per 1000 PY for those without TPT | 4 | 469 | 1388 | 38 | 65 (26-103) | 0.001 | 100% |
| Incidence rate difference | 3 | 868 | 2580 | 46 | 44 (20-94) | <0.001 | 0% |
|  |  |  |  |  |  |  |  |
| Incidence rate ratio | 3 | 868 | 2580 | 46 | 0.02 (0.00 - 0.89) # | 0.03 | 71.6% |

*TPT = tuberculosis preventive therapy.
 § = derived from random-effects meta-analysis; # incidence rate ratio is not expressed per 1000 PY, but as a ratio*

Supplementary table 7: numbers needed to screen to detect one case of LTBI or to prevent one case of TB disease

|  | Studies (n) | Participants (n) | Pooled LTBI prevalence (95% CI)^§^ | Number needed to screen to detect one case of LTBI† (95% CI) | Number needed to screen to prevent one case of active TB† (95% CI) |
| --- | --- | --- | --- | --- | --- |
| Overall population | 51 | 112,346 | 12% (10-14%) | 8 (8-8) | 167 (155-180) |
| Foreign born | 8 | 4,810 | 18% (13-24%) | 6 (5-6) | 111 (86-158) |
| Native-born | 5 | 14,339 | 7% (4-10%) | 14 (13-15) | 285 (224-395) |
| Origin from a TB endemic country | 11 | 2,582 | 15% (11-19%) | 7 (6-7) | 133 (92-240) |
| Sub-Saharan African # | 4 | 852 | 12% (7-17%) | 8 (7-10) | 167 (90-1228) |
| African-American # | 4 | 3,958 | 13 (5-21%) | 8 (8-8) | 154 (137-175) |
| Exposure (TB contact or previous TB exposure) | 4 | 140 | 24% (5-43%) | 4 (3-6) | 129 (45- 148) |
| Europe | 20 | 31,896 | 10% (8-12%) | 10 (10-10) | 200 (173-237) |
| North America | 28 | 78,541 | 13% (10-16) | 8 (8-8) | 154 (142-168) |
| Oceania | 3 | 1,909 | 9% (1-18%) | 11 (10-13) | 222 (133-668) |

### *§ = derived from random-effects meta-analysis*

*† = calculated using the number needed to treat of 31*
