# Supplementary material for: Screening for tuberculosis infection and effectiveness of preventive treatment among people with HIV in low-incidence settings
Source: AIDS. 2023 Nov 22;38(2):193–205. doi: 10.1097/QAD.0000000000003747 (PMC10734787; doi:10.1097/QAD.0000000000003747)
Supplement: Supplementary file 2 [file aids-38-193-s002.pdf]

Author	Setting	n	N	ES (95% CI)	% Weight
Europe					
Girardi (2000)	Italy	95	1360	0.07 (0.06, 0.08)	2.08
Antonucci (2001)	Italy	81	1215	0.07 (0.05, 0.08)	2.07
Brock (2006)	Denmark	27	590	0.05 (0.03, 0.07)	2.06
Diez (2007)	Spain	203	954	0.21 (0.19, 0.24)	2.01
Stephan (2008)	Germany	98	275	0.36 (0.30, 0.42)	1.75
Aichelburg (2009)	Austria	37	830	0.04 (0.03, 0.06)	2.07
Bua (2010)	Italy	8	73	0.11 (0.05, 0.20)	1.59
Kall (2012)	U.K.	46	520	0.09 (0.07, 0.12)	2.02
Cheallaigh (2013)	Ireland	46	256	0.18 (0.13, 0.23)	1.85
Martínez-Pino (2013)	Spain	428	2540	0.17 (0.15, 0.18)	2.07
Pullar (2014)	Norway	64	304	0.21 (0.17, 0.26)	1.86
Bourgarit (2015)	France	48	415	0.12 (0.09, 0.15)	1.98
Sester (2016)	Europe	122	768	0.16 (0.13, 0.19)	2.02
van Bentum (2018)	the Netherlands	29	599	0.05 (0.03, 0.07)	2.06
Cordioli (2018)	Italy	38	717	0.05 (0.04, 0.07)	2.06
Aston (2019)	U.K.	2	25	0.08 (0.01, 0.26)	1.23
Capocci (2020)	U.K.	14	217	0.06 (0.04, 0.11)	1.97
Goletti (2020)	Italy	32	495	0.06 (0.04, 0.09)	2.04
Kusejko (2020)	Switzerland	834	13675	0.06 (0.06, 0.07)	2.10
Sandhu (2020)	U.K.	3	128	0.02 (0.00, 0.07)	2.01
Subtotal (I^2 = 96.65%, p = 0.00)				0.11 (0.09, 0.13)	38.91
North America					
CDC (1993)	U.S.	287	1516	0.19 (0.17, 0.21)	2.05
Markowitz (1993)	U.S.	64	1171	0.05 (0.04, 0.07)	2.08
Huebner (1994)	U.S.	57	479	0.12 (0.09, 0.15)	1.99
Mofenson (1995)	U.S.	26	183	0.14 (0.09, 0.20)	1.81
Daley (1998)	U.S.	95	338	0.28 (0.23, 0.33)	1.84
Eriksen (1998)	U.S.	18	159	0.11 (0.07, 0.17)	1.82
Gampper (1998)	U.S.	34	341	0.10 (0.07, 0.14)	1.97
Gourevitch (1998)	U.S.	25	159	0.16 (0.10, 0.22)	1.75
Sackoff (1998)	U.S.	45	757	0.06 (0.04, 0.08)	2.06
Snyder (1998)	U.S.	111	472	0.24 (0.20, 0.28)	1.92
Anastos (1999)	U.S.	63	1343	0.05 (0.04, 0.06)	2.08
CDC (2000)	U.S.	12	95	0.13 (0.07, 0.21)	1.64
Narita (2002)	U.S.	178	2127	0.08 (0.07, 0.10)	2.08
Schulte (2002)	U.S.	54	207	0.26 (0.20, 0.33)	1.72
Lobato (2003)	U.S.	1863	4435	0.42 (0.41, 0.43)	2.07
Scholten (2003)	U.S.	141	373	0.38 (0.33, 0.43)	1.82
Lee (2006)	U.S.	58	869	0.07 (0.05, 0.09)	2.06
Luetkemeyer (2007)	U.S.	36	294	0.12 (0.09, 0.17)	1.93
Golub (2008)	U.S.	128	800	0.16 (0.14, 0.19)	2.02
Marks (2008)	U.S.	3	83	0.04 (0.01, 0.10)	1.91
Brassard (2009)	Canada	67	476	0.14 (0.11, 0.18)	1.98
Talati (2009)	U.S.	27	336	0.08 (0.05, 0.11)	1.99
Pascopella (2014)	U.S.	20	393	0.05 (0.03, 0.08)	2.04
Trieu (2015)	U.S.	5	167	0.03 (0.01, 0.07)	2.02
Shin (2016)	U.S.	813	15346	0.05 (0.05, 0.06)	2.10
Reaves (2017)	U.S.	131	1907	0.07 (0.06, 0.08)	2.08
Stout (2018)	U.S.	182	1366	0.13 (0.12, 0.15)	2.06
Ho (2022)	U.S.	155	1873	0.08 (0.07, 0.10)	2.08
Subtotal (I^2 = 99.09%, p = 0.00)				0.13 (0.10, 0.17)	54.99
Oceania					
Lyne (2013)	Australia	17	240	0.07 (0.04, 0.11)	1.97
Doyle (2014)	Australia	29	917	0.03 (0.02, 0.05)	2.08
Gow (2017)	New Zealand	74	752	0.10 (0.08, 0.12)	2.04
Subtotal (I^2 = .%, p = .)				0.07 (0.02, 0.11)	6.09
Heterogeneity between groups: p = 0.064					
Overall (I^2 = 98.61%, p = 0.00);				0.12 (0.10, 0.14)	100.00

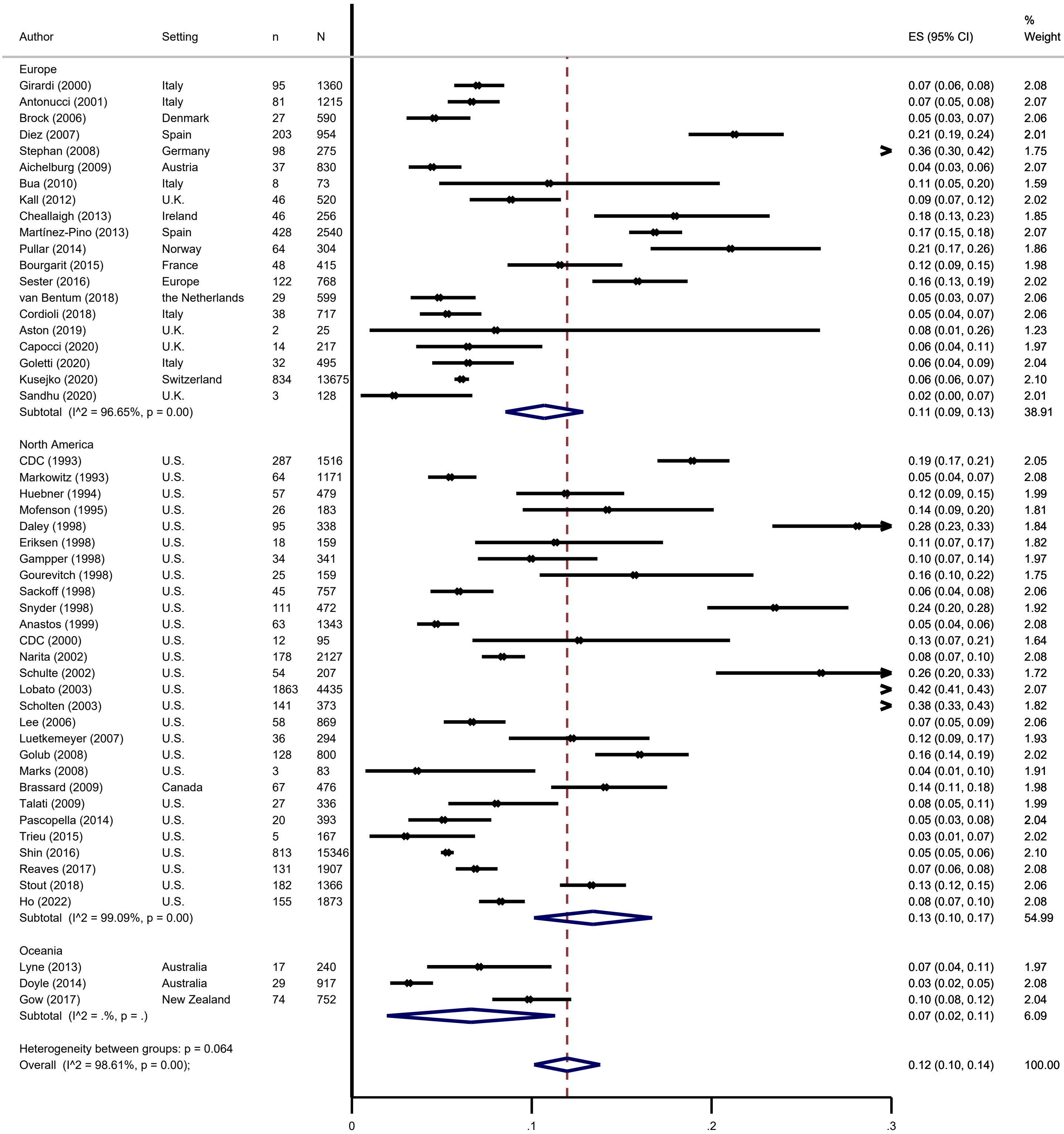

Supplementary figure 1 - Forest plot of LTBI-prevalence among PLHIV in low TB-incidence settings stratified by geographic region.
