# Supplementary figures and images for: Screening for tuberculosis infection and effectiveness of preventive treatment among people with HIV in low-incidence settings
Source: AIDS. 2023 Nov 22;38(2):193–205. doi: 10.1097/QAD.0000000000003747 (PMC10734787; doi:10.1097/QAD.0000000000003747)

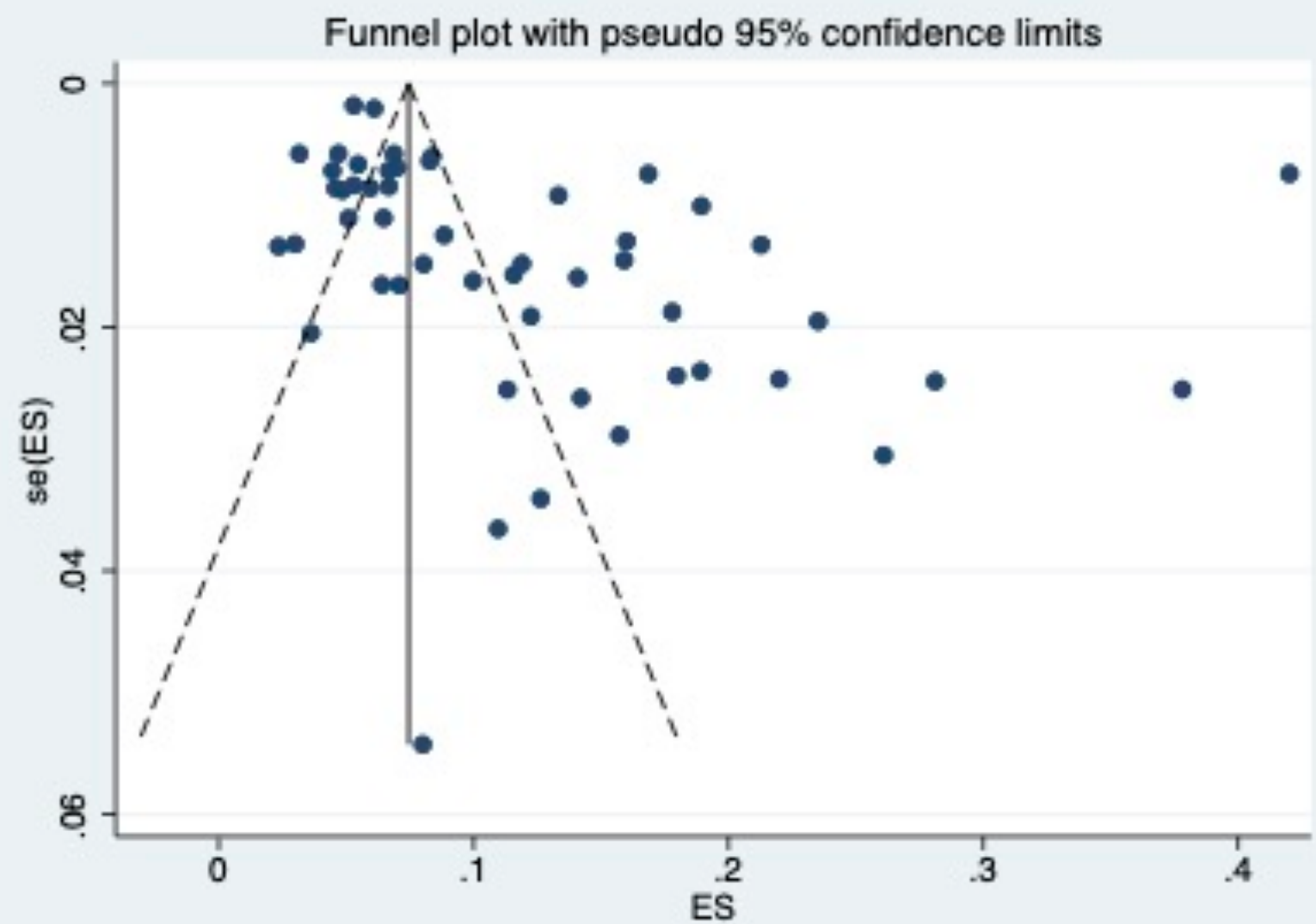

Supplementary figure 2 - Funnel plot showing evidence for small-study effects.

Supplement: Supplementary file 3 [file aids-38-193-s003.pdf]
